# Modal-Polar Representation of Evoked Response Potentials in Multiple Arousal States

**DOI:** 10.3389/fnhum.2021.642479

**Published:** 2021-06-07

**Authors:** Rawan K. El-Zghir, Natasha C. Gabay, Peter A. Robinson

**Affiliations:** ^1^School of Physics, University of Sydney, Sydney, NSW, Australia; ^2^Center for Integrative Brain Function, University of Sydney, Sydney, NSW, Australia

**Keywords:** neural field theory, brain dynamics, evoked response potentials, brain resonances, eigenmodes

## Abstract

An expansion of the corticothalamic transfer function into eigenmodes and resonant poles is used to derive a simple formula for evoked response potentials (ERPs) in various states of arousal. The transfer function corresponds to the cortical response to an external stimulus, which encodes all the information and properties of the linear system. This approach links experimental observations of resonances and characteristic timescales in brain activity with physically based neural field theory (NFT). The present work greatly simplifies the formula of the analytical ERP, and separates its spatial part (eigenmodes) from the temporal part (poles). Within this framework, calculations involve contour integrations that yield an explicit expression for ERPs. The dominant global mode is considered explicitly in more detail to study how the ERP varies with time in this mode and to illustrate the method. For each arousal state in sleep and wake, the resonances of the system are determined and it is found that five poles are sufficient to study the main dynamics of the system in waking eyes-open and eyes-closed states. Similarly, it is shown that six poles suffice to reproduce ERPs in rapid-eye movement sleep, sleep state 1, and sleep state 2 states, whereas just four poles suffice to reproduce the dynamics in slow wave sleep. Thus, six poles are sufficient to preserve the main global ERP dynamics of the system for all states of arousal. These six poles correspond to the dominant resonances of the system at slow-wave, alpha, and beta frequencies. These results provide the basis for simplified analytic treatment of brain dynamics and link observations more closely to theory.

## 1. Introduction

Evoked response potentials (ERPs) reflect the electrical activity of the brain triggered by sensory stimuli or events (Niedermeyer and Lopes Da Silva, 1999). ERPs have been widely used to provide windows on cognitive processes such as attention and perception (Luck and Kappenman, 2013). Most notably, attentional modulation enhances both early and late features of an ERP (Hillyard and Anllo-Vento, 1998; Herrmann and Knight, 2001; Vázquez Marrufo et al., 2001; Yamagishi et al., 2003). It has been shown that many characteristics of the stimulus and subject affect ERP waveforms (Picton et al., 2000; Womelsdorf et al., 2007; Woodman, 2010), notably including the state of arousal of the subject, with very different ERPs in sleep than wake, for example (Feng et al., 2012).

Traditional phenomenological analysis reduces ERPs to a small set of “components” defined by amplitudes and latencies (time delays after the stimulus) each of which correspond to a peak or trough in the waveform (Luck, 2014); each component is presumed to be generated by a group of excitatory or inhibitory neurons that have a certain cognitive role (Luck and Kappenman, 2013), but there is no explicit link to physiology and it is common to omit most data points and focus on only amplitudes and latencies of a few components. Moreover, it has been widely recognized that ERPs can be treated as impulse responses whose building blocks are damped sinusoids that reflect the dynamics of the underlying physical system that generates them (Kelly and Reilly, 1983).

Over the last 20 years, many quantitative brain studies have been performed based on a corticothalamic neural field theory (NFT) (Wright and Liley, 1996; Robinson et al., 1997, 2002, 2004, 2005). It has been shown that much brain activity is approximately linear and that ERPs can thus be described by a system transfer function that describes the response to a delta function input (Rennie et al., 1999; Robinson et al., 2005; Kerr et al., 2008). Such a function can also be used to calculate responses to arbitrary stimuli, and to derive other activity-dependent quantities such as correlations (Robinson et al., 2018; Robinson, 2019). Corticothalamic NFT averages over scales of a few tenths of a mm and has been an essential tool to accurately predict significant electroencephalographic (EEG) features such as ERPs that have been previously calculated and verified against experiment (Rennie et al., 1999; Robinson et al., 2005; Kerr et al., 2008). The corticothalamic model introduces physiologically based parameters that correspond to different physical quantities, and each real brain state is described by a particular set of parameters; consequently, transfer functions can be related to underlying physiology via NFT.

Within NFT, the transfer function can be expanded in terms of eigenfunctions which are the natural modes of the brain (Robinson et al., 1997, 2001) and the building blocks of normal brain dynamics (Pinotsis et al., 2013; Robinson et al., 2016). Eigenmodes of the corticothalamic system manifest on the cortical surface at length scales detectable with EEG, magnetoencephalography (MEG), and fMRI. Using NFT these modes can be described in terms of physiological parameters such as corticocortical, intrathalamic, and corticothalamic feedback loop strengths and inverse synaptodendritic decay and rise times (Gabay and Robinson, 2017). Such a modal perspective has yielded fruitful results regarding dynamic brain connectivity via spectral analysis (Gabay and Robinson, 2017; Gabay et al., 2018).

NFT expressions for the time dependence of activity are relatively complicated, as are the expressions for the transfer functions, which involve transcendental equations and are not analytically tractable. This poses a problem when comparing with experimental results that often involve expressions in terms of characteristic frequencies and time delays. Hence, it is desirable to recast the outcomes of NFT in terms of observable quantities in order to bridge more directly between theory and experiment. Recent work has begun to address this problem via rational function approximations to the temporal transfer function that express it compactly in terms of a small number of poles, as is often done in control theory (Babaie-Janvier and Robinson, 2018). Interestingly, this approach has yielded interpretations of EEG resonances in terms of proportional-integral-derivative (PID) filters that are commonly used in engineering control systems for predicting the future course of inputs within their corresponding frequency ranges (Kwakernaak and Sivan, 1972; Ogata, 2010; Babaie-Janvier and Robinson, 2019).

In the present work we use NFT to analyze cortical activity by representing the arousal states of the brain in terms of its normal modes. This approach has also been used to approximate brain activity by decomposing the transfer function first in terms of eigenfunctions (spatial modes) then the temporal response of each eigenfunction in terms of poles (resonant frequencies), but not many results about resonances are available in the literature, except for waking states of arousal (Gabay et al., 2018). In order to better understand how many poles to include for accurate representations, we systematically investigate the modal-polar representation of the transfer function in various states of arousal and provide parameters of the poles in each state for the first time. Hence, the present work provides a modal-polar representation of corticothalamic NFT that bridges between the mathematical NFT and the applied work done by neuroscientists in measuring resonances and impulse responses.

In this paper, the temporal transfer function is decomposed in terms of poles or resonances to derive a general formula for ERPs which greatly simplifies their analytical form. This framework simplifies NFT results, and links NFT to observables by expanding previous work that has calculated the poles for the waking states only (Gabay et al., 2018). It also enables observed resonances to be interpreted in terms of the transfer function's poles, and hence data filters of a type previously shown to implement prediction and attention (Babaie-Janvier and Robinson, 2018, 2019). Section 2 provides an overview of the corticothalamic NFT. In section 3, we illustrate the derivation of ERP formula based on modal-polar expansion. Then, in section 4 we show and discuss our results. Finally, section 5 summarizes our work.

## 2. Materials and Methods

In this section we first review the relevant corticothalamic NFT in section. 2.1, based on previous studies (Robinson et al., 1997, 2002; Kerr et al., 2008; Babaie-Janvier and Robinson, 2018; Gabay et al., 2018). Further details can be found in the references cited. Then, we use the modal-polar representation of the corticothalamic NFT as a tool for finding an explicit expression of the transcendental transfer function in terms of the brain's natural modes and their corresponding poles. Finally, in section 2.3 we discuss the spectral features and parameters characterizing each arousal state.

### 2.1. Corticothalamic Neural Field Theory

**Figure 1:**
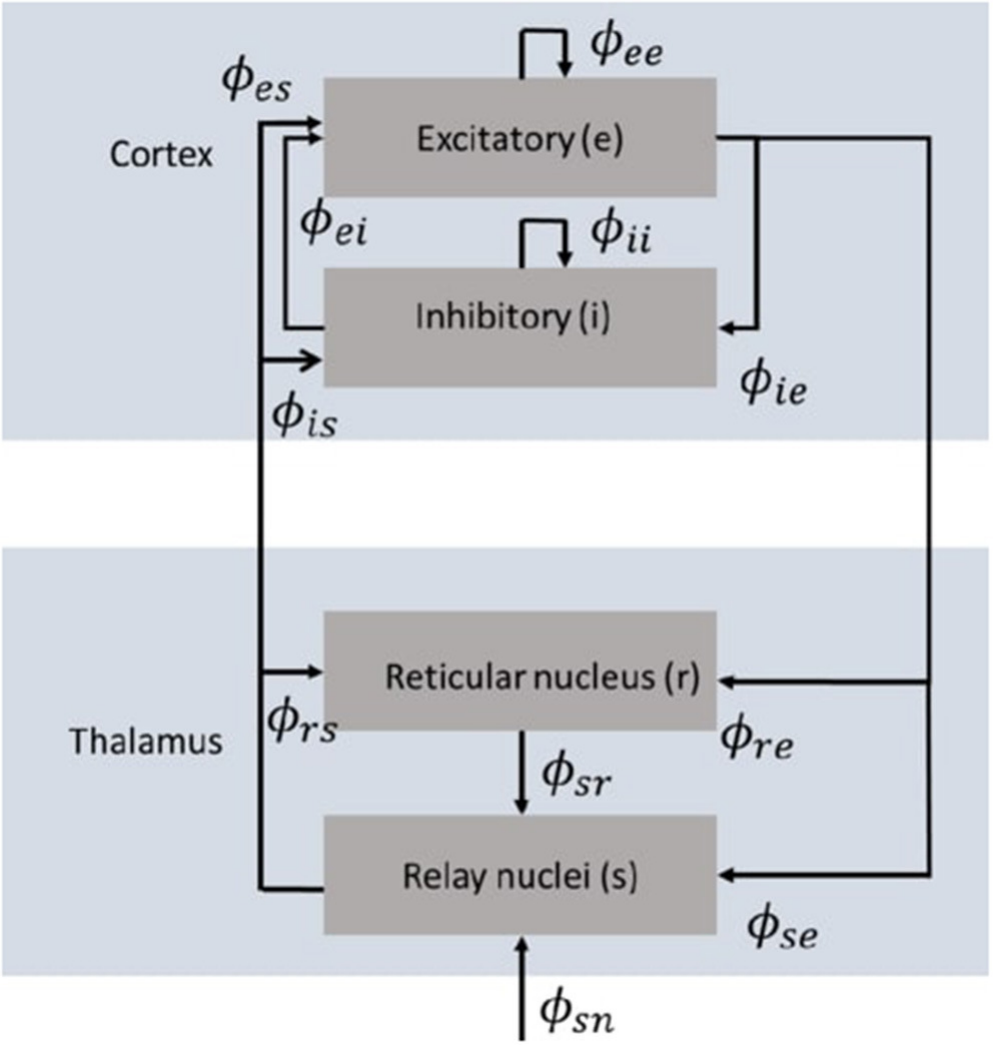
Schematic diagram of the corticothalamic model (Gabay and Robinson, 2017) that incorporates key anatomic connectivities between neural populations, where ϕ_*ab*_ is the mean activity field reaching population *a* due to signals from population *b*. There are two cortical populations of excitatory (*e*) and inhibitory (*i*) neurons, and two thalamic populations corresponding to the thalamic reticular nucleus (*r*) and the thalamic relay nuclei (*s*).

Neural field theory averages neural quantities over scales of a few tenths of a millimeter. It has been widely used to interpret and reproduce key features of experimental findings in EEG and fMRI. The neural field model consists of cortical excitatory (*e*) and inhibitory (*i*) populations, thalamic specific relay populations (*s*), thalamic reticular populations (*r*), and external sensory inputs (*n*). This model incorporates key anatomic connectivities between those populations, as shown in Figure 1, where ϕ_*ab*_ is the mean activity field reaching population *a* due to signals from population *b*. The strength of connection to population *a* from population *b* is Robinson et al. (2005)

(1) $$\begin{array}{l} \nu_{ab}=s_{ab}N_{ab}, \end{array}$$

where *s*_*ab*_ is the mean time-integrated strength of the response in neurons *a* per incoming signal from neurons *b*, and *N*_*ab*_ is the mean number of synapses to neurons *a* from *b*.

It is found that population *a*'s average firing rate *Q*_*a*_ is linked to the corresponding average membrane potential *V*_*a*_, relative to resting, by a nonlinear sigmoid function

(2) $$\begin{array}{l} Q_{a}=S\left(V_{a}\right)=\frac{Q_{\max}}{1+\exp\left[-\left(V_{a}-\theta \right)/\sigma^{\prime }\right]}, \end{array}$$

where *Q*_max_ is the maximum firing rate, θ is the mean threshold voltage, and $\sigma^{\prime }\pi /\sqrt{3}$ is the standard deviation of the threshold distribution.

Due to synaptodendritic dynamics and soma capacitance, presynaptic inputs *b* from different types of neurons *a* are summed after being filtered and smeared out in time, giving rise to the potential *V*_*a*_ such that

(3) $$\begin{array}{l} V_{a}\left(\text{r},t\right)=\underset{b}{\sum }V_{ab}\left(\text{r},t\right). \end{array}$$

We can convert from the signal/stimulus to the population response using the following equation:

(4) $$\begin{array}{l} D_{\alpha }\left(t\right)V_{ab}\left(\text{r},t\right)=\nu_{ab}\phi_{ab}\left(\text{r},t-\tau_{ab}\right), \end{array}$$

where *D*_α_ is the wave propagation operator, given by

(5) $$\begin{array}{l} D_{\alpha }\left(t\right)=\frac{1}{\alpha \beta }\frac{d^{2}}{dt^{2}}+\left(\frac{1}{\alpha }+\frac{1}{\beta }\right)\frac{d}{dt}+1, \end{array}$$

where **r** is the position vector on the brain, 1/β and 1/α are the rise and decay times, respectively, of the potential at the cell body elicited by an impulse response at the dendritic synapse, and τ_*ab*_ is the time delay due to anatomical separations between neural populations *a* and *b*, as specified in Table 1. The only nonzero time delays correspond to propagation time from cortex to thalamus and vice versa (τ_*ab*_ ≈ 0 in the case of intrathalamic and intracortical connections).

**Table 1:** Nominal parameters of corticothalamic neural field theory based on previous work (Babaie-Janvier and Robinson, 2019).

**Symbol**	**Quantity**	**Value**	**Units**
α	Synaptodendritic decay rate	80	s^−1^
β	Synaptodendritic rise rate	320	s^−1^
τ_*es*_	Thalamocortical axonal delay	0.02	s
τ_*se*_	Corticothalamic relay axonal delay	0.06	s
γ_*ee*_	Cortical damping rate	116	s^−1^
*r*_*ee*_	Excitatory axon range	0.086	m

The mapping of the source firing rates *Q*_*b*_(**r**, *t*) into the axonal signal is achieved within the following equations (Robinson et al., 1997)

(6) $$\begin{array}{l} D_{ab}\left(\text{r},t\right)\phi_{ab}\left(\text{r},t\right)=Q_{b}\left(\text{r},t\right), \end{array}$$

(7) $$\begin{array}{l} D_{ab}\left(\text{r},t\right)=\frac{1}{\gamma_{ab}^{2}}\frac{\partial^{2}}{\partial t^{2}}+\frac{2}{\gamma_{ab}}\frac{\partial }{\partial t}+1-r_{ab}^{2}\nabla^{2}, \end{array}$$

which describes the propagation of a mean activity field ϕ_*ab*_(_**r**_,*t*) that obeys a damped wave equation where γ_*ab*_ = *v*_*ab*_/*r*_*ab*_ is the temporal damping rate, *r*_*ab*_ is the mean characteristic range of axons to population *a* from population *b*, and *v*_*ab*_ is the propagation velocity in axons to population *a* from population *b*.

The mean axonal ranges for all populations except for excitatory cortical neurons are very short so we can write *r*_*ab*_ ≈ 0 for *b* = *i, r, s*, yielding *D*_*ab*_ = 1 for these neural populations. Let us assume that the number of intracortical synapses is proportional to the number of neurons involved, then *N*_*ib*_ = *N*_*eb*_ for all *b* (Wright and Liley, 1996; Robinson et al., 1997; Braitenberg and Schüz, 1998). We also assume that the input stimulus ϕ_*sn*_ is not too large such that the system has a fixed point with low firing rates (Robinson et al., 2002). Hence, we can apply linear approximation where we henceforth treat each dynamic quantity (ϕ_*ab*_, *Q*_*a*_, *V*_*a*_) as a linear perturbation from its steady state value, which is denoted by the superscript (0). Then

(8) $$\begin{array}{l} Q_{a}\left(\text{r},t\right)=\rho_{a}V_{a}\left(\text{r},t\right), \end{array}$$

where

(9) $$\begin{array}{l} \rho_{a}=S^{\prime }\left(V_{a}^{\left(0\right)}\right), \end{array}$$

is the derivative with respect to voltage of the sigmoid function evaluated at the steady state.

By taking the Fourier transform of the above equations, we get

(10) $$\begin{array}{l} V_{ab}\left(\text{k},\omega \right)=L\left(\omega \right)\nu_{ab}\phi_{ab}\left(\text{k},\omega \right)e^{i\omega \tau_{ab}}, \end{array}$$

(11) $$\begin{array}{l} L\left(\omega \right)={\left(1-\frac{i\omega }{\alpha }\right)}^{-1}{\left(1-\frac{i\omega }{\beta }\right)}^{-1}, \end{array}$$

(12) $$\begin{array}{l} D_{ab}\left(\text{k},\omega \right)\phi_{ab}\left(\text{k},\omega \right)=Q_{b}\left(\text{k},\omega \right), \end{array}$$

where **k** is the wave vector and *L*(ω) embodies a low-pass filter response function. Activity *Q*_*b*_ generates fields of activity ϕ_*ab*_ that propagate through the brain to affect populations *a*. We express the firing rate ϕ_*ee*_ in terms of the external stimulus ϕ_*sn*_ via the transfer function

(13) $$\begin{array}{ll} T\left(\text{k},\omega \right) & =\frac{\phi_{ee}\left(\text{k},\omega \right)}{\phi_{sn}\left(\text{k},\omega \right)}, \end{array}$$

(14) $$\begin{array}{l} =\frac{L^{2}G_{es}G_{sn}e^{i\omega \tau_{es}}}{\left(1-L^{2}G_{sr}G_{rs}\right)\left(1-G_{ei}L\right)}\frac{1}{k^{2}r_{ee}^{2}+q^{2}\left(\omega \right)r_{ee}^{2}}, \end{array}$$

where we assign

(15) $$\begin{array}{l} q^{2}\left(\omega \right)r_{ee}^{2}={\left(1-\frac{i\omega }{\gamma_{ee}}\right)}^{2}\\ \;-\frac{1}{1-G_{ei}L}\left[LG_{ee}+\frac{L^{2}G_{es}\left(G_{se}+LG_{sr}G_{re}\right)\exp\left[i\omega \left(\tau_{es}+\tau_{se}\right)\right]}{1-L^{2}G_{sr}G_{rs}}\right]. \end{array}$$

and *G*_*ab*_ is the gain of responses in population *a* due to signals from population *b* such that *G*_*ab*_ = ρ_*a*_ν_*ab*_. The above form of the transfer function is transcendental, which is not easy to work with and does not link easily to observable features such as resonances in the EEG spectrum, so we seek to simplify it via the approximations below. Note that, in a linear system, all the other ϕ_*ab*_ are linearly related to ϕ_*ee*_.

Let $D^{\prime }\left(\omega \right)=q^{2}\left(\omega \right)r_{ee}^{2}$, then in the absence of external stimulus Equation (14) yields

(16) $$\begin{array}{l} D^{\prime }\left(\omega \right)\phi_{ee}=-r_{ee}^{2}\nabla^{2}\phi_{ee}, \end{array}$$

where ∇^2^ is the Laplace-Beltrami operator.

We analyze the spatiotemporal dynamics of the brain in terms of discrete modes labeled η. To solve Equation (16) for these eigenmodes, we introduce the ansatz

(17) $$\begin{array}{l} \phi_{ee}=u_{\eta }\left(\text{r}\right)e^{-i\omega_{\eta }t}, \end{array}$$

where *u*_η_(**r**) represents the spatial eigenmode on the cortical surface oscillating at an eigenfrequency ω_η_. Substituting (Equation 17) into (Equation 16) and using separation of variables yields the Helmholtz equation for the spatial eigenmode (Gabay and Robinson, 2017)

(18) $$\begin{array}{l} \nabla^{2}u_{\eta }\left(\text{r}\right)=-k_{\eta }^{2}u_{\eta }\left(\text{r}\right), \end{array}$$

where *u*_η_(**r**) are the eigenmode solutions of the equation, *k*_η_ are the wavenumbers and $k_{\eta }^{2}$ are the eigenvalues. By substituting the eigenvalues $k_{\eta }^{2}$ from Equation (18) into the dispersion relation

(19) $$\begin{array}{l} k_{\eta }^{2}+q_{\eta }^{2}\left(\omega \right)=0, \end{array}$$

we get

(20) $$\begin{array}{l} D_{\eta }^{\prime }\left(\omega \right)=-k_{\eta }^{2}r_{ee}^{2}. \end{array}$$

There are an infinite number of solutions of the dispersion relation because it is transcendental, although, as has been previously shown, a small number dominate the dynamics. These solutions correspond to the eigenfrequencies of the eigenmodes *u*_η_(**r**) and they correspond to the poles of the transfer function $T\left(k_{\eta }^{2},\omega \right)$ (Gabay et al., 2018).

### 2.2. Modal-Polar Expansion

The transfer function represents the cortical response to an external stimulus. It encodes all the properties of the linear system. By expanding the transfer function in terms of modes, we can decompose (Equations 13, 14) into two separate parts: the modal part which is spatial, and the temporal part, as in Equation (17). Separation of variables is more general, but if we assume that the transfer function is spatially symmetric, then the set of eigenmodes *u*_η_ is complete and orthonormal, which means that any connectivity or activity whatsoever can be expressed in terms of *u*_η_ (Robinson et al., 2018), as

(21) $$\begin{array}{l} T\left(\text{r},\text{r}\prime ,\omega \right)=\underset{\eta }{\sum }u_{\eta }\left(\text{r}\right)u_{\eta }^{*}\left(\text{r}\prime \right)\theta_{\eta }\left(\omega \right), \end{array}$$

where *u*_η_(**r**) are the eigenmodes, and θ_η_(ω) is the temporal part of the transfer function defined in Equation (23). Also, we assume that the brain is a static network on the timescales of interest, then the transfer function depends on the time difference *t* − *t*_0_ (Robinson, 2019); i.e.,

(22) $$\begin{array}{l} T\left(\text{r},t;{\text{r}}_{0},t_{0}\right)=T\left(\text{r},{\text{r}}_{0},t-t_{0}\right). \end{array}$$

Because the dispersion relation in Equation (20) is transcendental, it has an infinite number of solutions. This means that there are infinite number of poles (eigenfrequencies) for the corresponding eigenmode *u*_η_(**r**). However, transfer functions are ratios of exponential polynomials of −*iω*. Then, applying rational approximation enables us to write the transcendental transfer function in Equation (15) in the form (Gabay et al., 2018)

(23) $$\begin{array}{l} \theta_{\eta }\left(\omega \right)\approx \frac{\sum_{p=0}^{m}B_{p}\left(k_{\eta }^{2}\right){\left(-i\omega \right)}^{p}}{\sum_{q=0}^{n}A_{q}\left(k_{\eta }^{2}\right){\left(-i\omega \right)}^{q}}, \end{array}$$

the above rational approximation is Padé approximation of order (*m*/*n*) which is used to convert the transcendental transfer function into a rational polynomial (Equation 23), where *m* and *n* are the degrees of the numerator and denominator, respectively, and *n* > *m* (Golub and Van Loan, 1983). The coefficients *B*_*p*_ and *A*_*q*_ are real and depend on $k_{\eta }^{2}$ (the eigenvalue of the mode η), and *n* represents the number of poles where the denominator vanishes. Increasing accuracy is attained as we consider more poles in the expression. However, an important aim is to find the smallest number of poles that retains the main dynamics of the system while maintaining a high accuracy. Note that the form in Equation (23) has many advantages because of its analytic simplicity and pole structure (Gabay et al., 2018), as we see below.

Applying partial fraction decomposition of the rational approximate transfer function in Equation (23) yields (Varga, 1991; Babaie-Janvier and Robinson, 2018)

(24) $$\begin{array}{l} \theta_{\eta }\left(\omega \right)\approx \overset{n}{\underset{j=1}{\sum }}\frac{r_{\eta j}\left(k_{\eta }^{2}\right)}{\omega -\omega_{\eta j}\left(k_{\eta }^{2}\right)}, \end{array}$$

where *r*_η*j*_ and ω_η*j*_ are the residues and poles, respectively, and we truncate the sum at *j* = *n*. We assume that the modes are nondegenerate with wave numbers *k*_η_ and have complex eigenfrequencies ω_η*j*_. The resonant frequencies are of the form ω_η*j*_ = Ω_η*j*_ − *iγ*_η*j*_, where Ω_η*j*_ and γ_*j*_ are real and represent the angular frequency and damping rate, respectively, of the *j*^*th*^ pole at the η^*th*^ mode. We omit the explicit dependence on $k_{\eta }^{2}$, for simplicity, and write *T* for mode η as θ_η_(ω) in accordance with Equation (21). Then,

(25) $$\begin{array}{l} \theta_{\eta }\left(\omega \right)\approx \overset{n}{\underset{j=1}{\sum }}\frac{r_{\eta j}}{\omega -\omega_{\eta j}}. \end{array}$$

The form (21) with the above expression of θ_η_(ω) is termed modal-polar representation of corticothalamic transfer function.

### 2.3. Arousal State Characterization

In this section we apply our methods to arousal states that correspond to the levels defined by Rechtschaffen and Kales (1968): wake (W) which includes both the eyes-open (EO) and eye-closed (EC) states; sleep state 1 (S1) which refers to light sleep; sleep state 2 (S2) which is a deeper level of sleep; slow-wave sleep (SWS) which is the deepest level; rapid-eye movement (REM); and sleep spindles that Rechtschaffen and Kales (1968) defined them as “a burst of oscillatory brain activity visible on an EEG that occurs during S2.” In order to distinguish between each arousal state, we examine its corresponding EEG power spectrum. For normal adult humans, the frequency is conventionally divided into the following bands (Niedermeyer and Lopes Da Silva, 1999; Buzsáki and Draguhn, 2004): infraslow oscillations (0.01–0.1 Hz); delta (1.5–4 Hz); theta (4–7.5 Hz); alpha (7.5–13 Hz); spindle (11–16 Hz); and beta (13–30 Hz). The frequencies between 0.1 and 1.5 Hz are referred to as slow-waves, and they are characteristic waves in the delta band. Buzsáki and Draguhn (2004) divided these slow-wave frequencies into four classses (slow 1, slow 2, slow 3, and slow 4).

The waking EC state is distinguished by a distinctive strong alpha peak, whereas the waking EO state has a weaker alpha peak but similar alpha-band power (Rechtschaffen and Kales, 1968; Niedermeyer and Lopes Da Silva, 1999; Chiang et al., 2008). Eventually, as we move from the waking EC to S1, the alpha peak strength decreases significantly with an increase in the delta power, which continues to rise in the deep sleep states (Iber et al., 2007; Van Albada and Robinson, 2013).

Over the last 25 years, it has been shown that the changes in peak and trough timings and amplitudes are a natural consequence of the dominant EEG frequencies in the various states of arousal. In this paper, we are modeling what the impulse response would be in the various background EEG states, which is relevant if an external impulsive stimulus is applied, as in ERP experiments. This approach has been successfully used to model ERPs in multiple previous studies, however, the new technique in this framework is the use and verification that a few-pole approximation suffices to produce the same results, thereby leading to a more compact representation.

The responses of the system transfer functions for all populations are based on the nominal parameters shown in Tables 1, 2. The gains in Table 2 were derived by Abeysuriya et al. (2015) by fitting NFT predictions to experimental EEG spectra. For wake states, they used a data set of 2100 subjects with EEG recordings from the Brain Resource International Database, an archive of electrophysiological and psychophysiological measures, psychometric tests, and demographics (Gordon et al., 2005). For sleep states, they used manually scored polysomnograms from healthy controls (Wang et al., 2005; D'Rozario et al., 2013). These are spontaneous parameters and fits to observed ERPs could also be done using the same approach. Note that in Table 2, spindles are shown in one column with different gain parameters. Although spindles are considered to characterize Stage 2 sleep, previous findings suggest that spindles can be considered as transient substates and can span a different region of parameter space than simply S2 does (Abeysuriya et al., 2014). A state between SWS and Spindle would mix characteristics and produce a k-complex, a gallery of examples of these intermediate waveforms was shown in Figure 1 in Zobaer et al. (2017). Spindle dominates when that pole is only weakly damped; likewise, for slow waves. There is a region of parameter space where both poles are weakly damped and both oscillations can be seen. Figure 7 in Robinson et al. (2005) represents the stability zone and shows that the SWS and spindle zones meet. Gain parameters can in general be modulated by local feedback between populations in response to incoming stimuli, which has been useful for analyzing how evoked responses are shaped by attention (Babaie-Janvier and Robinson, 2020). Hence, we could tweak the parameters, but the core aim in this work is to determine how many poles are required to get a reasonable approximation to analytically predicted ERPs for the same parameters over the whole range of arousal states. Parameters could be changed to account for the fact that ERPs may be generated by a subsystem with somewhat different properties, for example (Robinson, 2005). However, the gains remain fixed in the present work which focuses on the theoretical ERP forms and their simplifications. However, the gains remain fixed in the present work which focuses on the theoretical ERP forms and their simplifications. It should be noted that these model parameters correspond to representative brain states from each of the sleep stages, where each sleep stage is associated with a range of parameters, reflecting individual differences in EEG between subjects at the same arousal stage (Abeysuriya et al., 2015).

**Table 2:** Gain parameters used in the present work based on previous studies from Abeysuriya et al. (2015) for the EO, EC, REM, S1, S2, SWS, and Spindles arousal states.

**Gain**	**EO**	**EC**	**REM**	**S1**	**S2**	**SWS**	**Spindles**
*G*_*ee*_	10.50	2.07	5.87	7.45	16.86	19.52	18.52
−*G*_*ei*_	13.22	4.11	6.61	8.30	17.93	19.74	18.96
*G*_*es*_	1.21	0.77	0.21	0.31	3.89	5.30	2.55
*G*_*se*_	5.78	7.77	0.66	1.67	0.07	0.22	0.73
−*G*_*sr*_	2.83	3.30	0.28	0.40	0.14	0.22	0.26
*G*_*sn*_	14.23	8.10	0.68	3.90	2.38	1.70	2.78
*G*_*re*_	0.85	0.66	2.08	7.47	4.96	1.90	4.67
*G*_*rs*_	0.25	0.20	4.59	4.44	8.33	1.35	16.92

## 3. Results

This section contains two sets of results: new theoretical developments and application to states of arousal. In the first part, we derive a simple expression for the ERP based on the modal-polar transfer function. In the second part, we apply the results to the different arousal states by plotting the locations of poles for different cases of number of poles and finding the root mean square error to study the convergence of *T*(*f*) (where the frequency in Hz is *f* = ω/2π) and ERP(*t*) to their exact results. This enables us to estimate the number of poles needed to attain high accuracy in studying each arousal state.

### 3.1. ERP Derivation

For simplicity, throughout the present work we restrict attention to the spatially uniform global mode ($k_{\eta }^{2}=0$), because this mode has the lowest damping rate and is the least stable, so it is the most easily excited and dominates the response (Robinson, 2003; Gabay and Robinson, 2017).

The ERP for a stimulus at (**r**_0_, *t*_0_) is identical to the transfer function; i.e.,

(26) $$\begin{array}{l} \text{ERP}\left(\text{r},{\text{r}}_{0},t\right)=T\left(\text{r},{\text{r}}_{0},t-t_{0}\right). \end{array}$$

If we let *t*_0_ = 0 without loss of generality and Fourier transform, then

(27) $$\begin{array}{l} \text{ERP}\left(\text{r},{\text{r}}_{0},\omega \right)=T\left(\text{r},{\text{r}}_{0},\omega \right), \end{array}$$

so

(28) $$\begin{array}{l} \text{ERP}\left(\text{r},{\text{r}}_{0},\omega \right)\approx \underset{\eta }{\sum }u_{\eta }\left(\text{r}\right)u_{\eta }^{*}\left({\text{r}}_{0}\right)\overset{n}{\underset{j=1}{\sum }}\frac{r_{\eta j}}{\omega -\omega_{\eta j}}. \end{array}$$

Applying an inverse Fourier transform we get

(29) $$\begin{array}{l} \text{ERP}\left(\text{r},{\text{r}}_{0},t\right)=\underset{\eta }{\sum }u_{\eta }\left(\text{r}\right)u_{\eta }^{*}\left({\text{r}}_{0}\right)\theta_{\eta }\left(t\right), \end{array}$$

(30) $$\begin{array}{l} \text{ERP}\left(\text{r},{\text{r}}_{0},t\right)\approx \underset{\eta }{\sum }u_{\eta }\left(\text{r}\right)u_{\eta }^{*}\left({\text{r}}_{0}\right)\overset{n}{\underset{j=1}{\sum }}\int \frac{d\omega }{2\pi }e^{-i\omega t}\frac{r_{\eta j}}{\omega -\omega_{\eta j}}, \end{array}$$

where

(31) $$\begin{array}{l} \theta_{\eta }\left(t\right)\approx \underset{j=1^{n}}{\sum }\theta_{\eta j}\left(t\right)=\overset{n}{\underset{j=1}{\sum }}\int \frac{d\omega }{2\pi }e^{-i\omega t}\frac{r_{\eta j}}{\omega -\omega_{\eta j}}, \end{array}$$

is the temporal part of the ERP due to the mode η.

#### 3.1.1. Integration Contour

**Figure 2:**
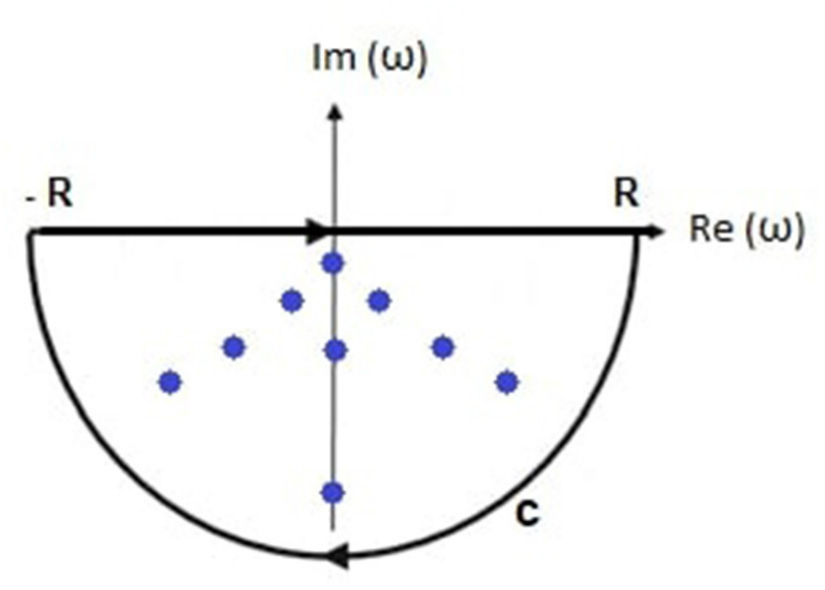
Schematic of the transfer function poles (dots) within the contour of integration comprising the real axis between −*R* and *R* and the semicircular arc C.

In this section, we evaluate Equation (31) by contour integration. The damping rate must be real and positive in a stable system, so Im(ω_η*j*_) < 0, as shown in Figure 2, and the appropriate integration contour lies in the negative imaginary half-plane of complex angular frequency and encloses all *n* poles being considered. Then

(32) $$\begin{array}{l} \oint \frac{d\omega }{2\pi }e^{-i\omega t}\frac{r_{\eta j}}{\omega -\omega_{\eta j}}=\underset{R\to \infty }{\lim}\int_{-R}^{R}\frac{dx}{2\pi }e^{-ixt}\frac{Re\left(r_{\eta j}\right)}{x-Re\left(\omega_{\eta j}\right)}\\ \;+\underset{R\to \infty }{\lim}\int_{C}\frac{d\omega }{2\pi }e^{-i\omega t}\frac{r_{\eta j}}{\omega -\omega_{\eta j}}. \end{array}$$

By Jordan's Lemma (Mitrinović, 1984),

(33) $$\begin{array}{l} \underset{R\to \infty }{\lim}\int_{C}\frac{d\omega }{2\pi }e^{-i\omega t}\frac{r_{\eta j}}{\omega -\omega_{\eta j}}=0. \end{array}$$

So

(34) $$\begin{array}{l} \oint \frac{d\omega }{2\pi }e^{-i\omega t}\frac{r_{\eta j}}{\omega -\omega_{\eta j}}=\underset{R\to \infty }{\lim}\int_{-R}^{R}\frac{dx}{2\pi }e^{-ixt}\frac{Re\left(r_{\eta j}\right)}{x-Re\left(\omega_{\eta j}\right)}. \end{array}$$

#### 3.1.2. Cauchy Residue Theorem

The integrand of θ_η*j*_(*t*) is analytic except at the frequencies ω_η*j*_, and the integral is over the closed contour in Figure 2, Cauchy's residue theorem then implies

(35) $$\begin{array}{l} \oint \frac{d\omega }{2\pi }e^{-i\omega t}\frac{r_{\eta j}}{\omega -\omega_{\eta j}}=-i\underset{\omega_{\eta j}}{\sum }\text{Res}\left[\frac{r_{\eta j}e^{-i\omega t}}{\omega -\omega_{\eta j}}\right], \end{array}$$

where Res denotes the residue of the function in brackets in Equation (35) and the sum is over all *n* poles.

For nondegenerate modes, there are only simple poles at ω = ω_η*j*_, and the residue is

(36) $$\begin{array}{l} \underset{\omega \to \omega_{\eta j}}{\lim}\left(\omega -\omega_{\eta j}\right)\frac{r_{\eta j}\;e^{-i\omega t}}{\omega -\omega_{\eta j}}=r_{\eta j}\;e^{-i\omega_{\eta j}t}. \end{array}$$

Remember that we have been setting *t*_0_ = 0, and writing *t* instead of *t* − *t*_0_ when evaluating the temporal part of the ERP. Thus, the general form of the temporal part of the ERP for arbitrary *t*_0_ is

(37) $$\begin{array}{l} \theta_{\eta j}\left(t\right)\approx -i\overset{n}{\underset{j=1}{\sum }}r_{\eta j}e^{-i\omega_{\eta j}\left(t-t_{0}\right)}=-i\overset{n}{\underset{j=1}{\sum }}r_{\eta j}e^{-i\Omega_{\eta j}\left(t-t_{0}\right)}e^{-i\gamma_{\eta j}}, \end{array}$$

for *t* ⩾ *t*_0_, and θ_η*j*_(*t*) = 0 for *t* < *t*_0_ to ensure causality, where ω_η*j*_, Ω_η*j*_, and γ_η*j*_ are defined in section 2.2.

#### 3.1.3. Pairing Up Poles

**Figure 3:**
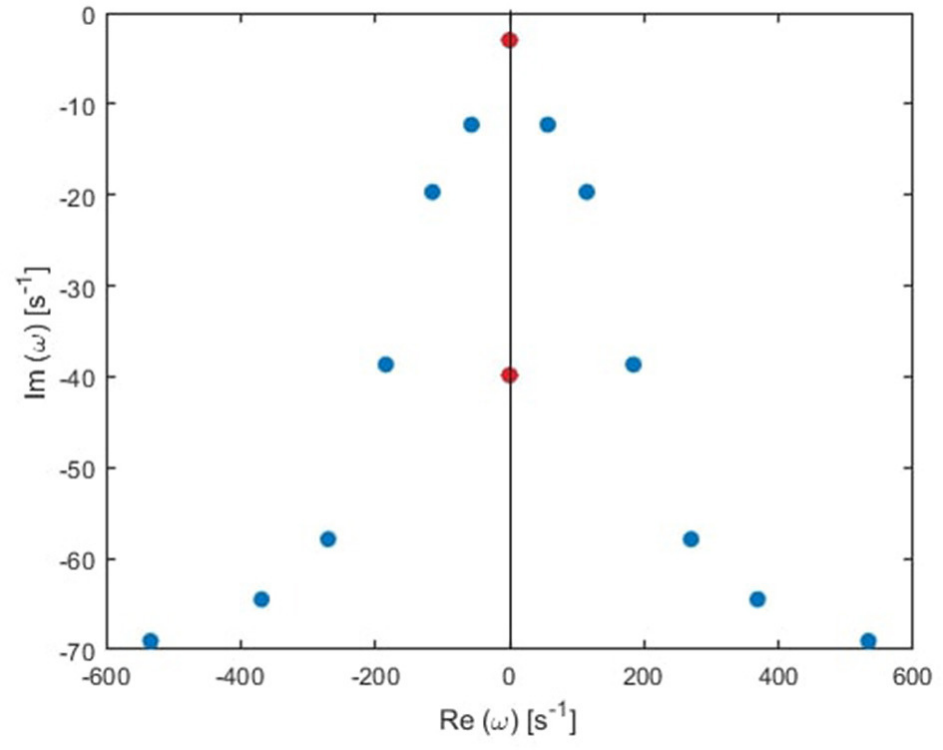
Transfer function poles (dots) in the Fourier space for the global mode in the human waking state. Blue and red dots correspond to oscillatory poles and purely damped poles, respectively.

In general, the poles of a transfer function can be grouped in pairs, such that each pair generates a real response in the time domain (Babaie-Janvier and Robinson, 2018). There are two types of pairs: the first type has two poles with the same damping rate (γ_η*j*_) and equal but opposite natural frequency (Ω_η*j*_), and the second type consists of two pure imaginary poles; paired for mathematical convenience. For simplicity, we call the first type oscillatory poles, and the second type purely damped, as illustrated in Figure 3. Accordingly, the temporal part of the ERP is

(38) $$\begin{array}{l} \theta_{\eta }\left(t\right)=\underset{O}{\sum }I_{\eta O}\left(t\right)+\underset{D}{\sum }Q_{\eta D}\left(t\right), \end{array}$$

where *I*_η*O*_(*t*) is the sum of θ_η_(*t*) over a single pair of oscillatory poles labeled *o* and *o*′ such that *O* = [*o, o*′], and *Q*_η*D*_(*t*) to a pair of purely damped poles labeled *d* and *d*′ such that *D* = [*d, d*′]. Note that the total number of poles *n* should be even in order to group them in pairs in our derivation. In the case when we retain an odd number of poles all the poles are paired except one purely damped pole.

To calculate *I*_η*O*_(*t*), we apply the reality condition (Howell, 2001; Babaie-Janvier and Robinson, 2018)

(39) $$\begin{array}{l} \omega_{\eta o^{\prime }}=-\omega_{\eta o}^{*}, \end{array}$$

and get

(40) $$\begin{array}{l} \omega_{\eta o}=\Omega_{\eta o}-i\gamma_{\eta o}, \end{array}$$

(41) $$\begin{array}{l} \omega_{\eta o^{\prime }}=-\Omega_{\eta o}-i\gamma_{\eta o}. \end{array}$$

Then

(42) $$\begin{array}{l} I_{\eta O}\left(t\right)=-ie^{-\gamma_{\eta o}t}\left[r_{\eta o}e^{-i\Omega_{\eta o}t}+r_{\eta o^{\prime }}e^{i\Omega_{\eta o}t}\right]. \end{array}$$

Also, we have

(43) $$\begin{array}{l} r_{\eta o^{\prime }}=-r_{\eta o}^{*}, \end{array}$$

and can write

(44) $$\begin{array}{l} r_{\eta o}=|r_{\eta o}|e^{-i\psi_{\eta o}}, \end{array}$$

(45) $$\begin{array}{l} \tan\left(\psi_{\eta o}\right)=\frac{\text{Im}\left(r_{\eta o}\right)}{\text{Re}\left(r_{\eta o}\right)}, \end{array}$$

where ψ_η*o*_ is the complex argument of *r*_η*o*_, so

(46) $$\begin{array}{ll} I_{\eta O}\left(t\right) & =ie^{-\gamma_{\eta o}t}|r_{\eta o}|\left[e^{i\left(\psi_{\eta o}+\Omega_{\eta o}t\right)}-e^{-i\left(\psi_{\eta o}+\Omega_{\eta o}t\right)}\right], \end{array}$$

(47) $$\begin{array}{l} =-2|r_{\eta o}|e^{-\gamma_{\eta o}t}\sin\left(\Omega_{\eta o}t+\psi_{\eta o}\right), \end{array}$$

which is a real response, as required.

Now consider the case of purely damped modes with

$$\begin{array}{l} \Omega_{\eta j}=0. \end{array}$$

Then

(48) $$\begin{array}{l} \omega_{\eta d}=-i\gamma_{\eta d}, \end{array}$$

(49) $$\begin{array}{l} \omega_{\eta d^{\prime }}=-i\gamma_{\eta d^{\prime }}. \end{array}$$

So

(50) $$\begin{array}{l} Q_{\eta D}\left(t\right)=-i\left(r_{\eta d}e^{-\gamma_{\eta d}t}+r_{\eta d^{\prime }}e^{-\gamma_{\eta d^{\prime }}t}\right). \end{array}$$

Note that *r*_η*d*_ and $r_{\eta d^{\prime }}$ are pure imaginary here, so

(51) $$\begin{array}{l} Q_{\eta D}\left(t\right)=\text{Im}\left(r_{\eta d}\right)e^{-\gamma_{\eta d}t}+\text{Im}\left(r_{\eta d^{\prime }}\right)e^{-\gamma_{\eta d^{\prime }}t}, \end{array}$$

which is also a real response. Finally, by combining the above results, we find

(52) $$\begin{array}{l} \text{ERP}\left(\text{r},{\text{r}}_{0},t\right)=\underset{\eta }{\sum }u_{\eta }\left(\text{r}\right)u_{\eta }^{*}\left({\text{r}}_{0}\right)\left[\underset{O}{\sum }I_{\eta O}\left(t\right)+\underset{D}{\sum }Q_{\eta D}\left(t\right)\right]. \end{array}$$

This equation expresses the ERP via a modal-polar representation of the transfer function. Each term has a spatial part $u_{\eta }\left(\text{r}\right)u_{\eta }^{*}\left({\text{r}}_{0}\right)$ and a temporal factor in the square brackets. Remember that we have been considering an even number of oscillatory and purely damped poles in the temporal part.

### 3.2. Pole Locations

**Figure 4:**
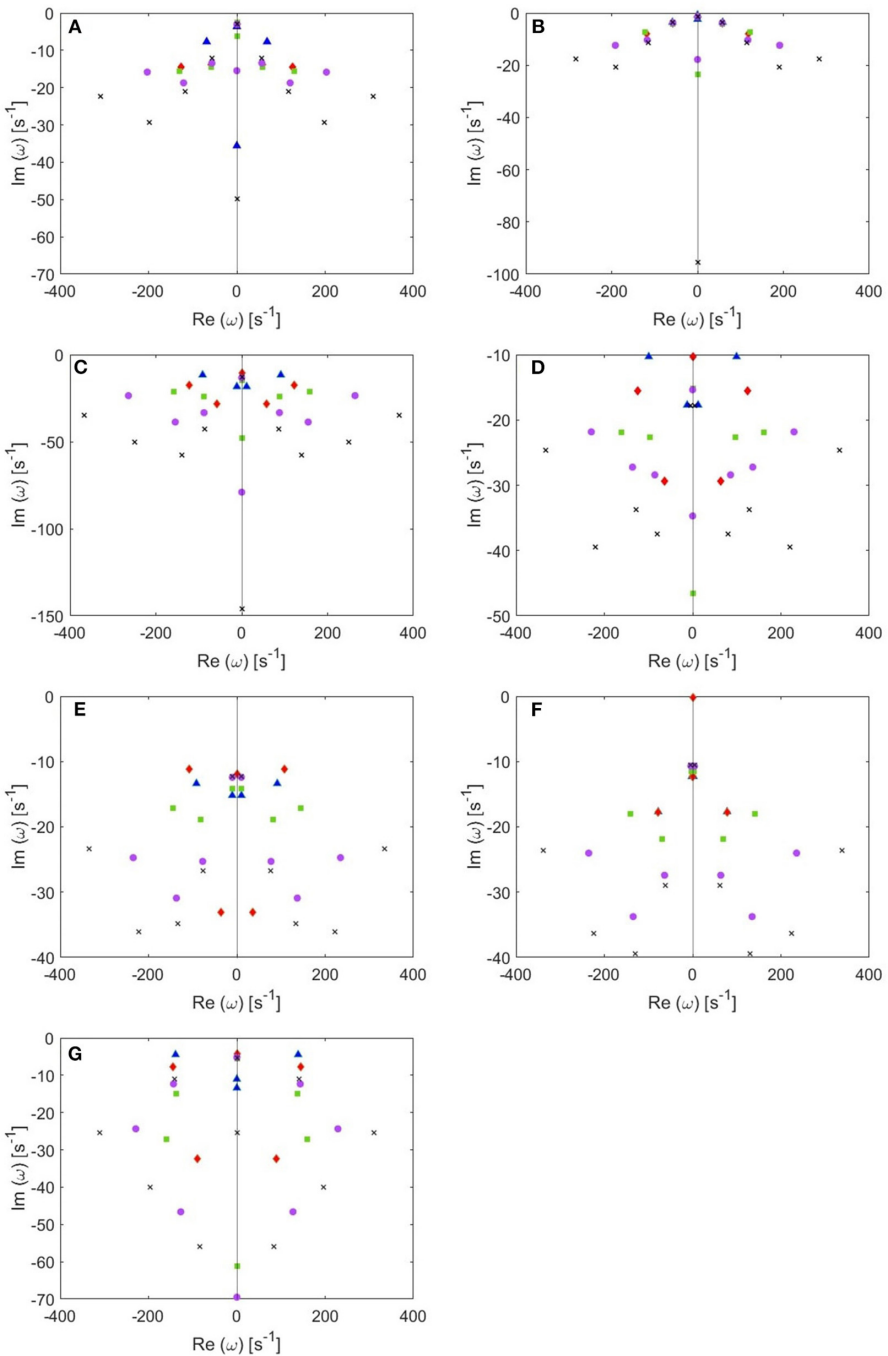
Location of poles for 4-pole (blue triangles), 5-pole (red rhombuses), 6-pole (green squares), 8-pole (purple circles), and 10-pole (black crosses) approximations to the transfer function. **(A)** EO state, **(B)** EC state, **(C)** REM state, **(D)** S1 state, **(E)** S2 state, **(F)** SWS state, and **(G)** Spindles state.

**Figure 5:**
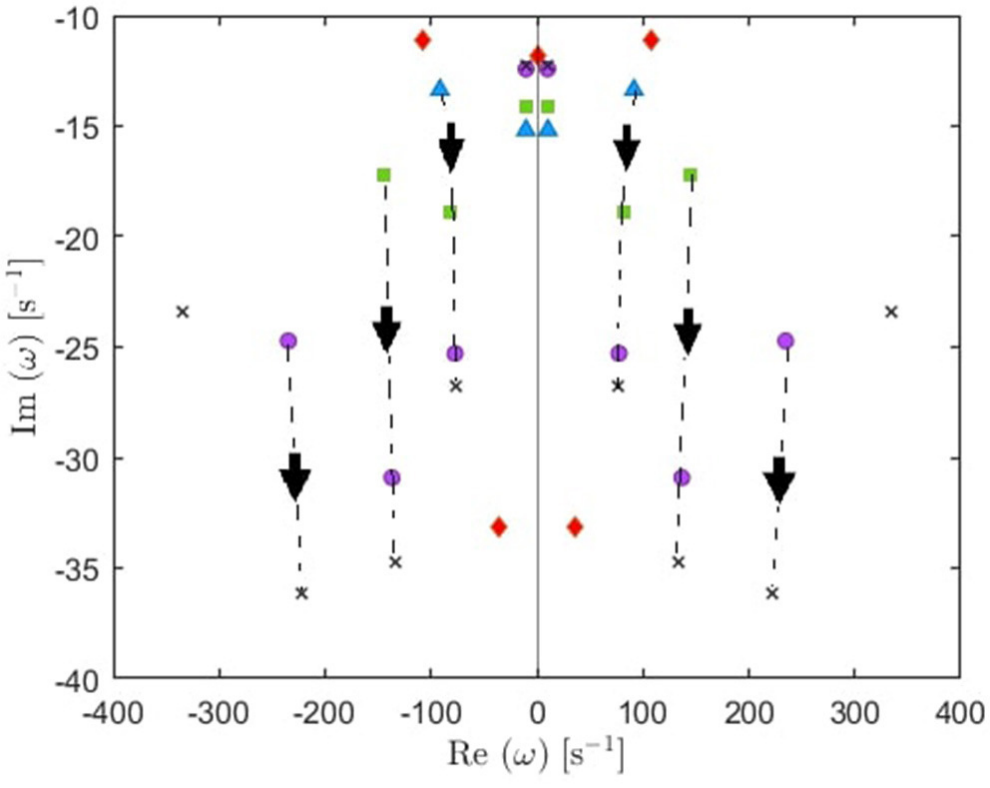
Enlarged view of Figure 4E showing how poles move as we add more poles to the approximation. Starting with 4 poles (blue triangles), we track (by arrows) how the oscillatory poles in the right and left of the figure symmetrically move when changing to 5-pole approximation (red rhombuses), 6-pole approximation (green squares), 8-pole approximation (purple circles), and 10-pole approximation (black crosses).

**Figure 6:**
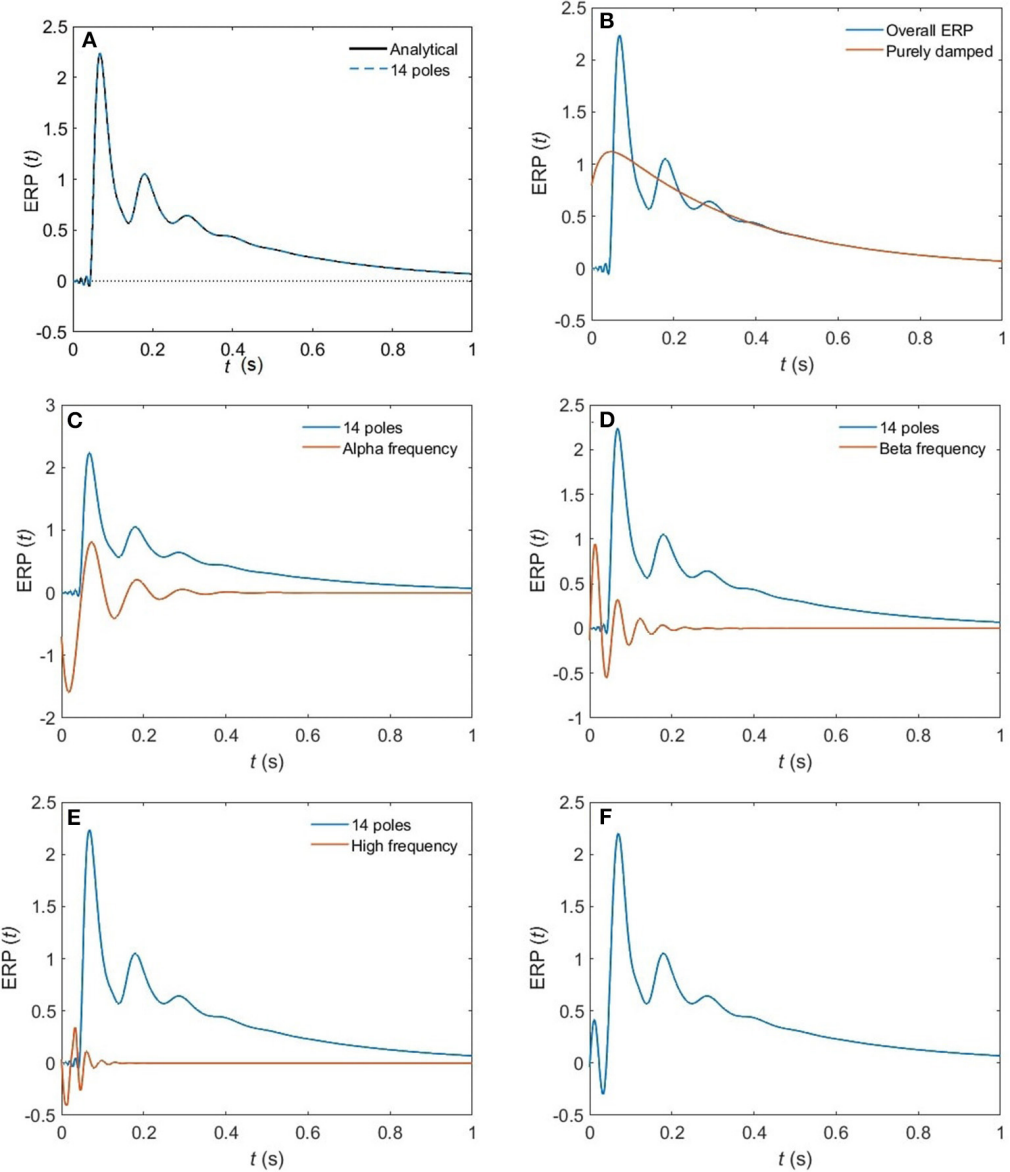
The change of shape of a 14-pole approximated ERP in the EO state upon only considering poles at specific frequencies. **(A)** Analytical ERP vs. 14-pole approximated ERP **(B)** purely damped poles. **(C)** Alpha-frequency poles. **(D)** Beta-frequency poles. **(E)** High-frequency poles (>30 Hz). **(F)** Removing high-frequency poles.

**Figure 7:**
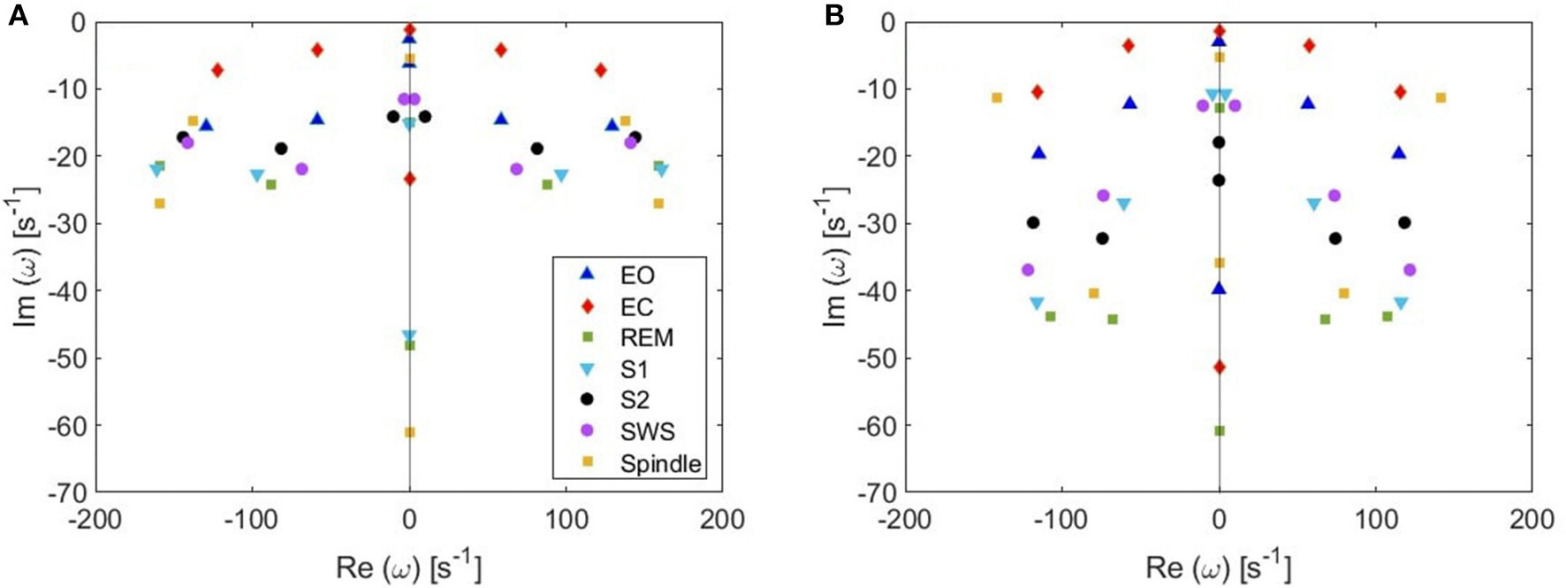
Pole locations in different arousal states. **(A)** 6-pole approximation. **(B)** 6 most weakly damped poles out of 14.

The locations of poles for several cases of the total number of poles are shown in Figure 4. Remember that the eigenfrequencies are of the form ω = Ω − *iγ*, where Ω corresponds to the angular frequency and γ to the damping rate. Our main results are:

- From these plots we figure out that as more poles are added, a given root usually moves downwards and approaches a limiting location (arrows in Figure 5), adding at most a few weakly damped poles; adding more poles in the analysis adds only strongly damped poles, including some at increasingly high frequencies. However, these poles contribute little to the overall dynamics in which the least damped roots are the dominant resonances. Because just a few weakly damped poles dominate the dynamics, addition of further poles only modifies the behavior slightly. Note that a very few poles move slightly upwards when we add more poles, as what happened to the beta frequency poles when changing from 4 to 5-pole approximation, but the general trend is moving downwards. Also, in Figure 5, we notice that some of the purely damped poles split into two oscillatory poles (red low frequency purely damped pole in a 5-pole approximation splits into two green oscillatory poles after changing to 6-pole approximation) and vice versa (two blue low frequency oscillatory poles in a 4-pole approximation add together to become one red purely damped pole after changing to 5-pole approximation).
- In the EO (Figure 4A) and EC (Figure 4B) waking states, the roots corresponding to alpha frequency shift slightly downwards as we add more poles, unlike the remaining sleep states (Figures 4C–F) that show a larger shift of the alpha roots as we add more poles. This is due to the need to reproduce the prominent alpha peak in the wake states, especially the EC state. We verify that by plotting the locations of poles of the Spindle condition in Figure 4G which shows that the poles corresponding to the spindle peak (at about 14 Hz) also stabilize at a limiting location like what happened in the waking states near their alpha and beta peak frequencies. This result is expected because it is well known in spectral analysis that whenever a peak exists, the damping rate is low (inversely proportional). Therefore, the distribution of poles (Figure 4) is a characteristic of the arousal state.
- Except for the least damped poles, the roots move rapidly toward large damping for the other sleep states as we increase the number of poles in the approximation, especially in the REM and S1 states. This outcome is predictable because REM and S1 states are classified as being the lightest sleep states, and their smooth spectra are fairly featureless, lack sharp peaks, and flatten at low frequencies. Therefore, these states only need the lowest ω = 0 poles to show their main dynamics.
- These poles enable us to plot ERPs for the different arousal states. Figure 6 shows the shape of a 14-pole approximated ERP function upon only considering poles at specific frequencies in the EO state. In Figure 6A, we show the close match between analytical ERP and the 14-pole approximated ERP. The 14-pole approximated ERP starts from zero with the appearance of very short and fast ripples before the stimulus hits the cortex (at τ_*es*_ = 0.02 ms), which then disappear and a sudden peak of the ERP takes place followed by consecutive smaller peaks decreasing in magnitude ending with an asymptotic decay toward zero after around 0.5 s. The ERP obtained by considering only purely damped poles (Ω_η*j*_ = 0), shown in Figure 6B, captures the asymptotic tail of the overall ERP and starts from a nonzero positive component, whereas the contribution of the alpha pole is negative (Figure 6C) at first. The ERPs formed by both the beta and high frequency poles (>30 Hz) show small peaks before the stimulus reaches the cortex (Figures 6D,E), and these small peaks disappear form the ERP if we remove the high frequency poles (Figure 6F). Therefore, when we add all poles together we get the ERP with very small residual ripples. This is analogous to ripples near a step change using finite number of Fourier coefficients. In real ERP analysis this is insignificant because there is always noise in the data.
- Because from Figure 5 we deduce that the most weakly damped poles dominate, we plot in Figure 7 the location of poles in a 6-pole approximation (Figure 7A), and the remaining 6 poles out of 14-pole approximation (Figure 7B) to remove highly damped poles across all the states of arousal. Both figures look similar for the EO and EC waking states except for small shifts in the frequencies of the poles. However, in the other sleep states, poles have stronger damping in Figure 7B than Figure 7A especially in the REM and S1 states. In general, the two figures are consistent with each other which verifies that the three most weakly damped pairs of poles dominate, and correspond to the slow-wave, alpha, and beta resonances. Note that the 6-pole approximation figure is better (Figure 7A), and it can also be used as a reference to examine the shift between sleep states according to their poles' locations.

### 3.3. Accuracy vs. Number of Poles

The model parameters identifying brain states representing each of the Rechtschaffen and Kales sleep states are shown in Table 2, where we use them to evaluate the model transfer function. Here we apply our modal-polar approximation in frequency space to the transfer function corresponding to each arousal state separately in order to test our model in multiple arousal states. To check the accuracy of our approximation, we calculate the normalized root mean square error (rms) according to

(53) $$\begin{array}{l} \epsilon =\int \frac{\sqrt{\bar{{\left(\left|T|-|T_{app}\right|\right)}^{2}}}}{\sqrt{{\bar{\left|T\right|}}^{2}}}d\omega . \end{array}$$

In the above rms error formula, we are considering the magnitude difference between the analytical and approximated transfer functions. The phase shift will not lead to much of a difference in ERPs because (at least in part) the magnitude of the high frequency components is very small. This is because the damping in the system attenuates high frequencies so the system only responds weakly to rapidly changing inputs. We could also use the Akaike information criterion (AIC) to choose the best fit on the basis of balancing accuracy against model complexity (i.e., number of poles in the present context) (Bozdogan, 1987). However, in the present work our aim is to achieve a given percentage accuracy, whereas AIC might trade off a poorer accuracy in favor of having fewer parameters.

**Figure 8:**
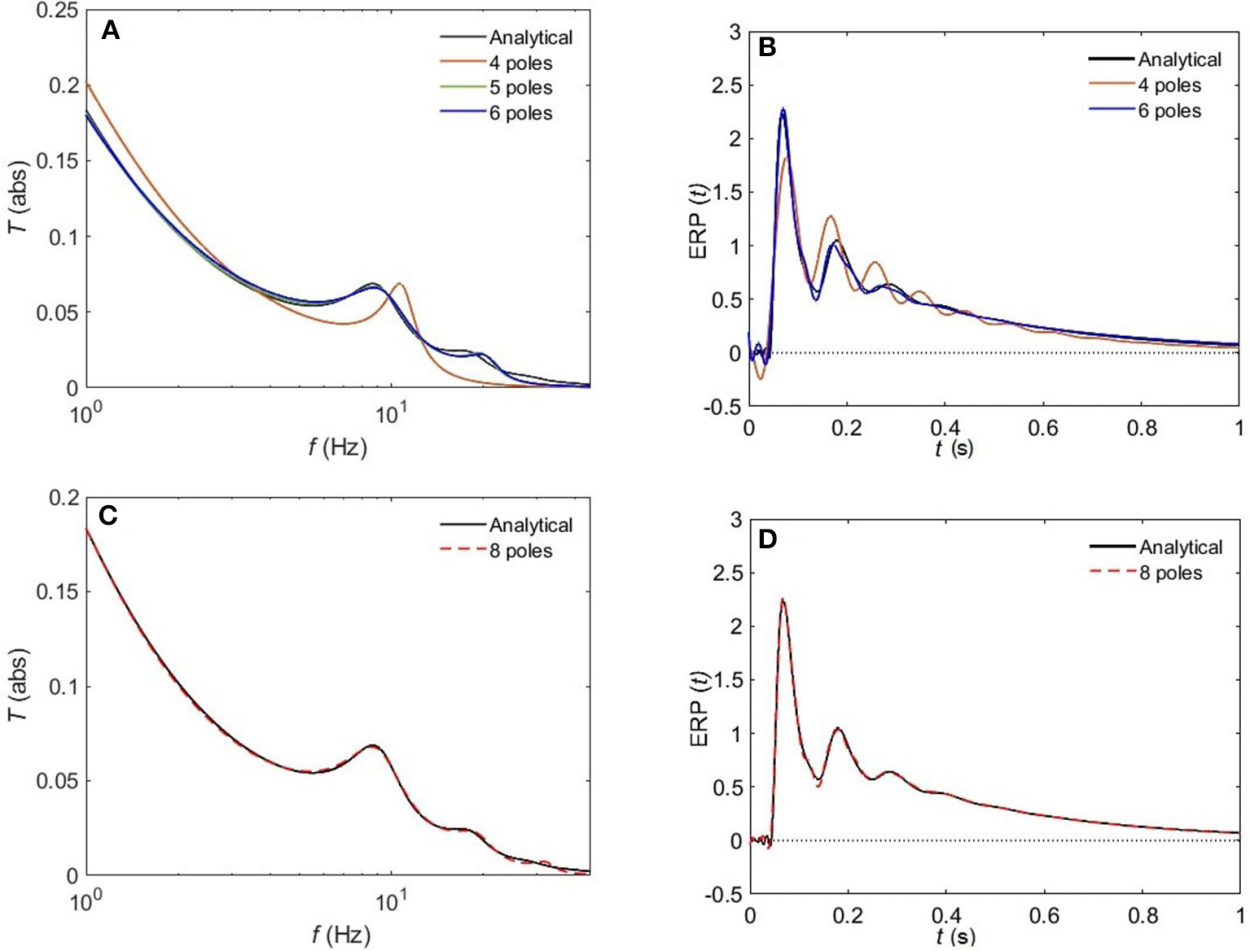
Comparison of magnitude of the analytical frequency and time responses of the transfer functions with their approximations in the EO waking state for different cases of number of poles. **(A)** Magnitude of the transfer function in the case of 4-pole approximation (red), 5-pole approximation (green), and 6-pole approximation (blue). **(B)** Magnitude of the evoked response potential in the case of 4-pole approximation (red), 5-pole approximation (green), and 6-pole approximation (blue). **(C)** Same as **(A)** for 8-pole approximation. **(D)** Same as **(B)** for 8-pole approximation. Polar approximated transfer functions' figures are plotted by evaluating Equation (25) vs. frequency, and ERPs' ones are obtained by evaluating Equation (38) vs. time.

**Figure 9:**
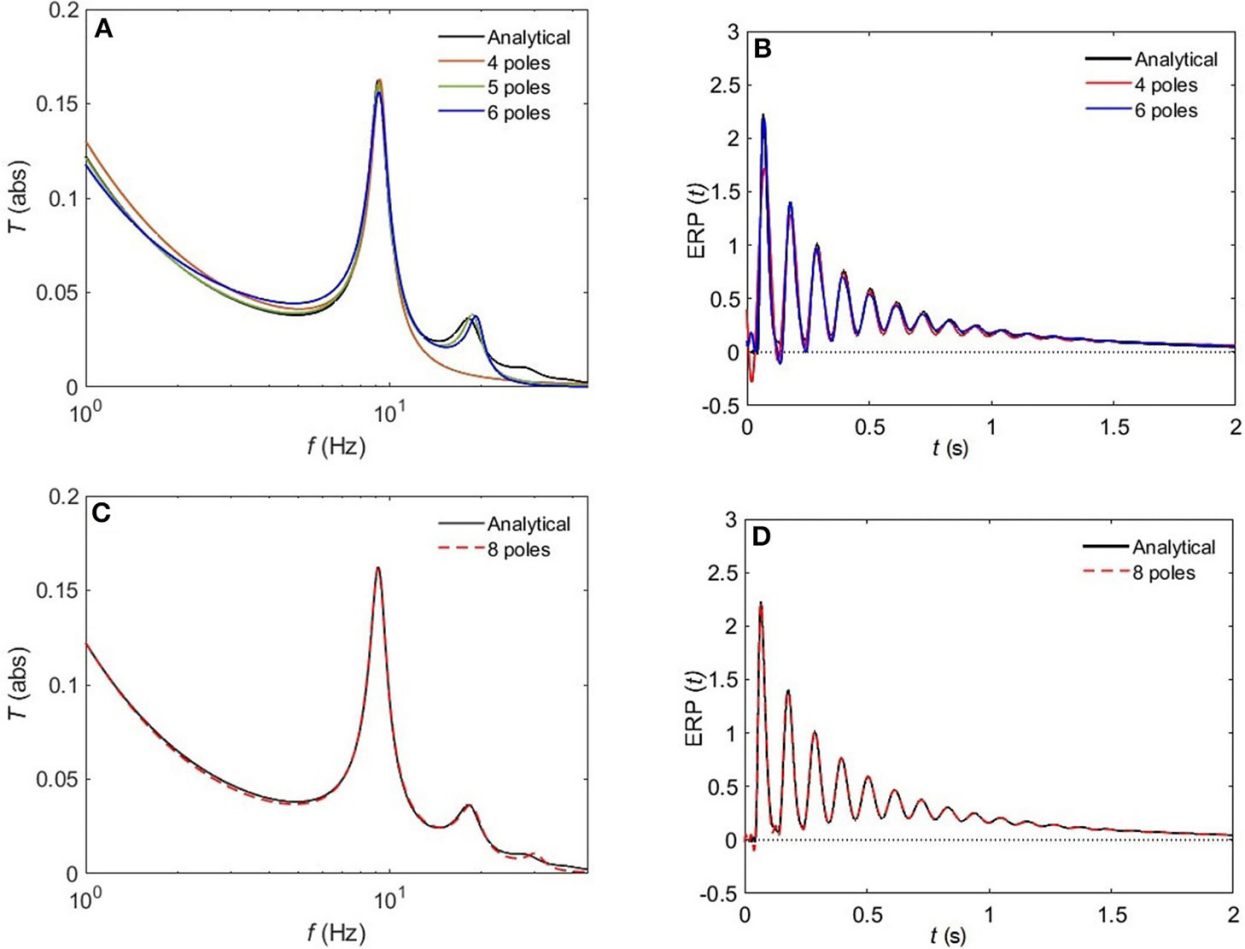
Comparison of magnitude of the analytical frequency and time responses of the transfer functions with their approximations in the EC waking state for different cases of number of poles. **(A)** Magnitude of the transfer function in the case of 4-pole approximation (red) and 5-pole approximation (green), and 6-pole approximation (blue). **(B)** Magnitude of the evoked response potential in the case of 4-pole approximation (red), 5-pole approximation (green), and 6-pole approximation (blue). **(C)** same as **(A)** for 8-pole approximation. **(D)** same as **(B)** for 8-pole approximation.

Figure 8 compares the approximated and analytical transfer functions (left) and their corresponding ERPs (right). In the alert EO state, as shown in Figure 8A, the analytical transfer function is characterized by possessing two consecutive peaks. The first peak corresponds to the alpha frequency (8.7 Hz), and the second one to the beta frequency (17.9 Hz). A 4-pole approximation of the EO transfer function yields to a large shift between the exact and approximated alpha peak in addition to missing the beta contribution. As we increase the number of poles to five, we observe a refined result, preserving the features of the transfer function with ϵ ≈ 4%. As we keep on adding poles to the approximation the results become more accurate, with about 1% error achieved by using eight poles (Figure 8C). In Figure 9, we repeat the same strategy for the EC waking state which is characterized by a prominent alpha peak followed by a beta peak. Similarly, four poles are insufficient to represent the corresponding transfer function accurately. However, five poles yield about 5% error, and ϵ < 2% for an 8-pole approximation.

**Figure 10:**
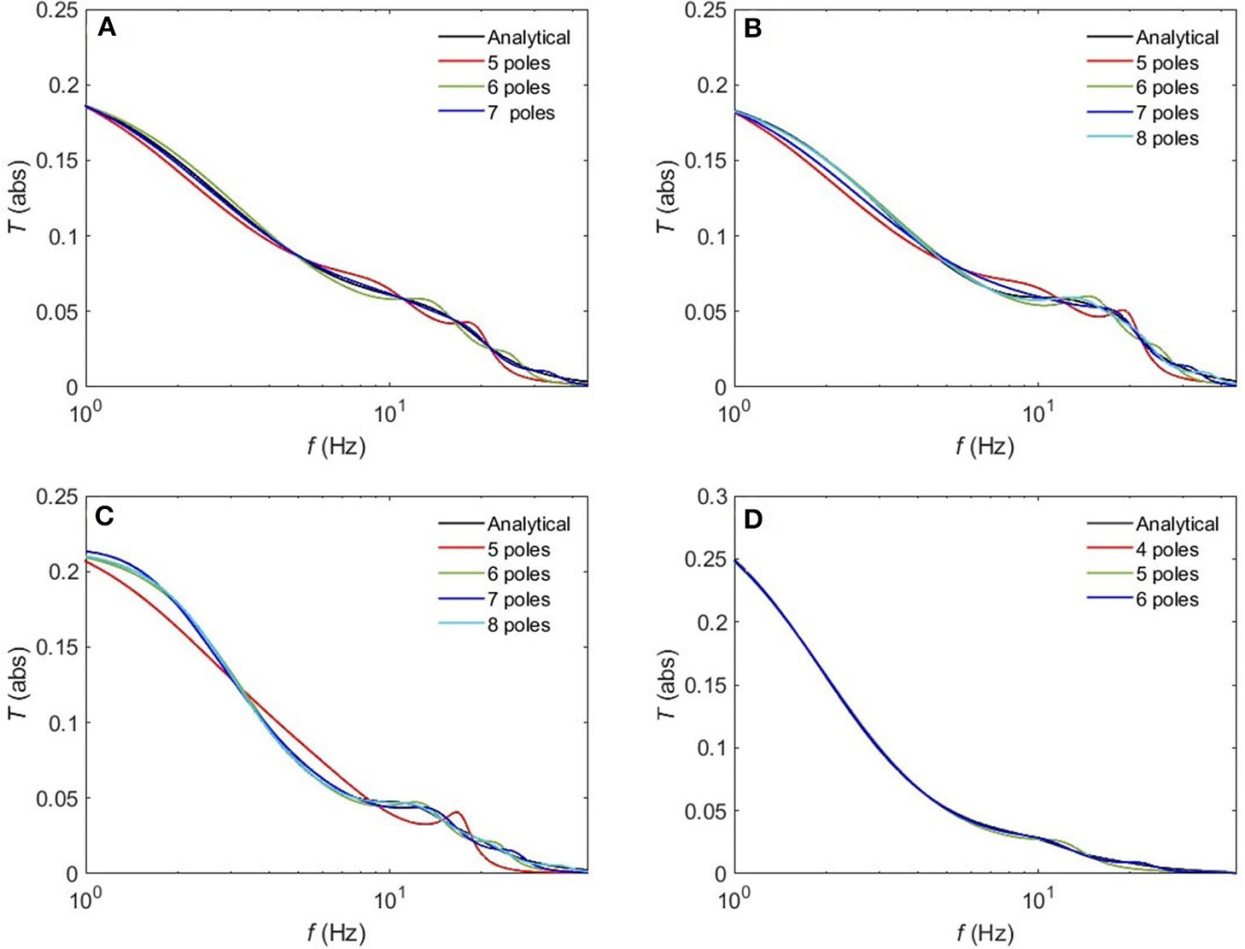
Comparison of the magnitude of the analytical transfer function with its polar approximation for sleep states. **(A)** REM, **(B)** S1, **(C)** S2, and **(D)** SWS.

**Figure 11:**
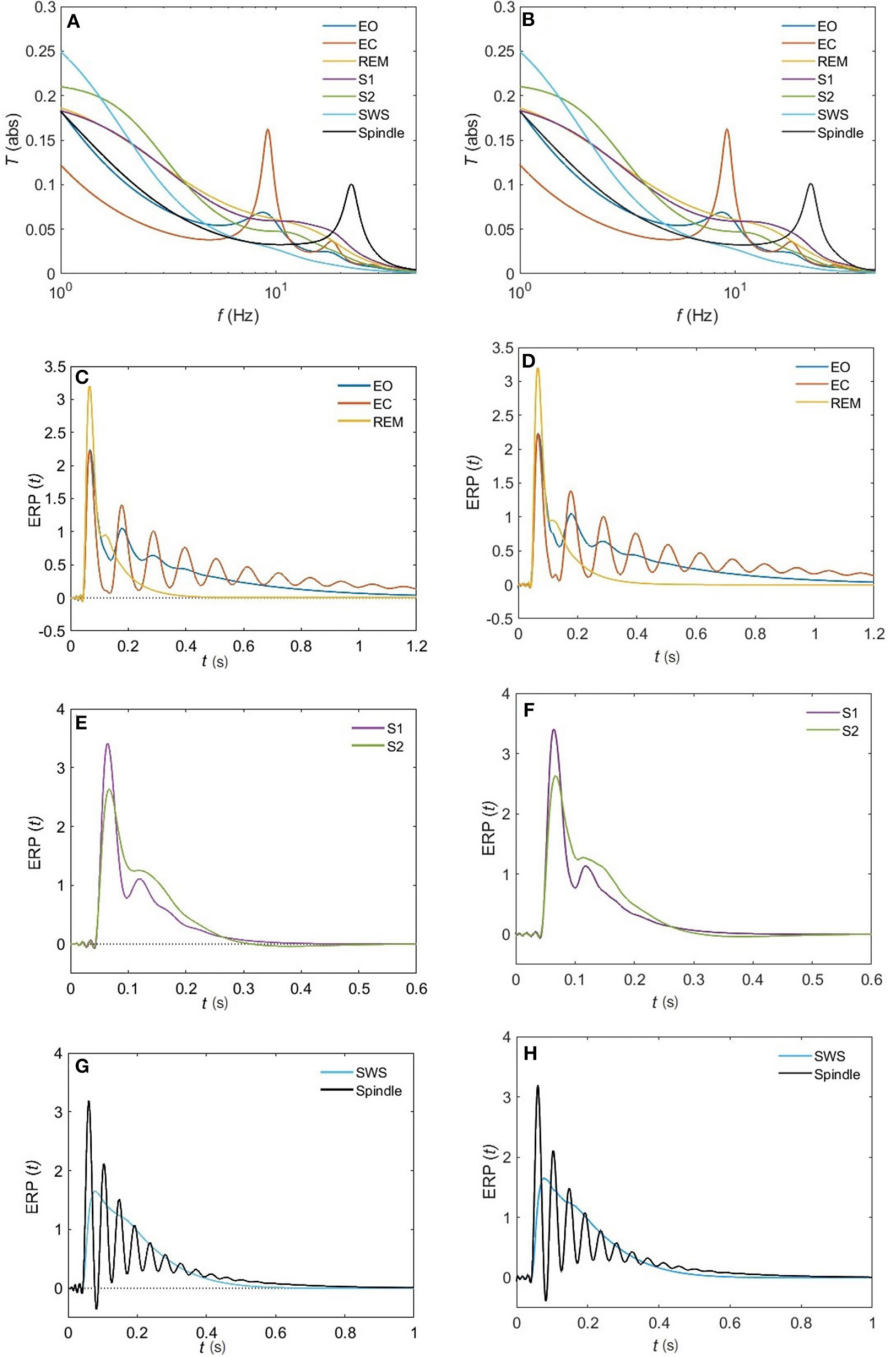
Comparison of the magnitude of the analytical transfer functions and ERPs with their 10-pole approximations for all the states of arousal. **(A)** Analytical transfer function. **(B)** 10-pole approximated transfer function. **(C)** Analytical ERP for the EO, EC, and REM states. **(D)** 10-pole approximated ERP for the EO, EC, and REM states. **(E)** Analytical ERP for the S1 and S2 states. **(F)** 10-pole approximated ERP for the S1 and S2 states. **(G)** Analytical ERP for the SWS and Spindle states. **(H)** 10-pole approximated ERP for the SWS and Spindle states.

The normalized rms error ϵ is worse for the sleep states (REM, S1, S2) compared to the waking states upon using a 5-pole approximation, with only about 10% error. However, Figure 10 reveals that a 6-pole approximation reproduces acceptable results for the REM, S1 and S2 states (ϵ <5%); but, we notice that there is a weak additional peak near the beta frequency which is absent in the corresponding analytical transfer functions. Adding one more pole cancels that extra peak in the REM state and leads to further enhancement of the transfer function with about 2% error, whereas, for the S1 and S2 states, two more poles are needed to attain a better resolution, with 2 and 0.8% error, respectively. For the deepest sleep state SWS in Figure 10D, the 4-pole approximation shows a very close match with the analytical SWS transfer function, with about 4% error. By increasing the number of poles to six, <2% error is achieved. Figure 11 shows a close match (ϵ < 1%) between the analytical and polar representation of both the transfer function and ERPs for all arousal states in a 10-pole approximation.

**Figure 12:**
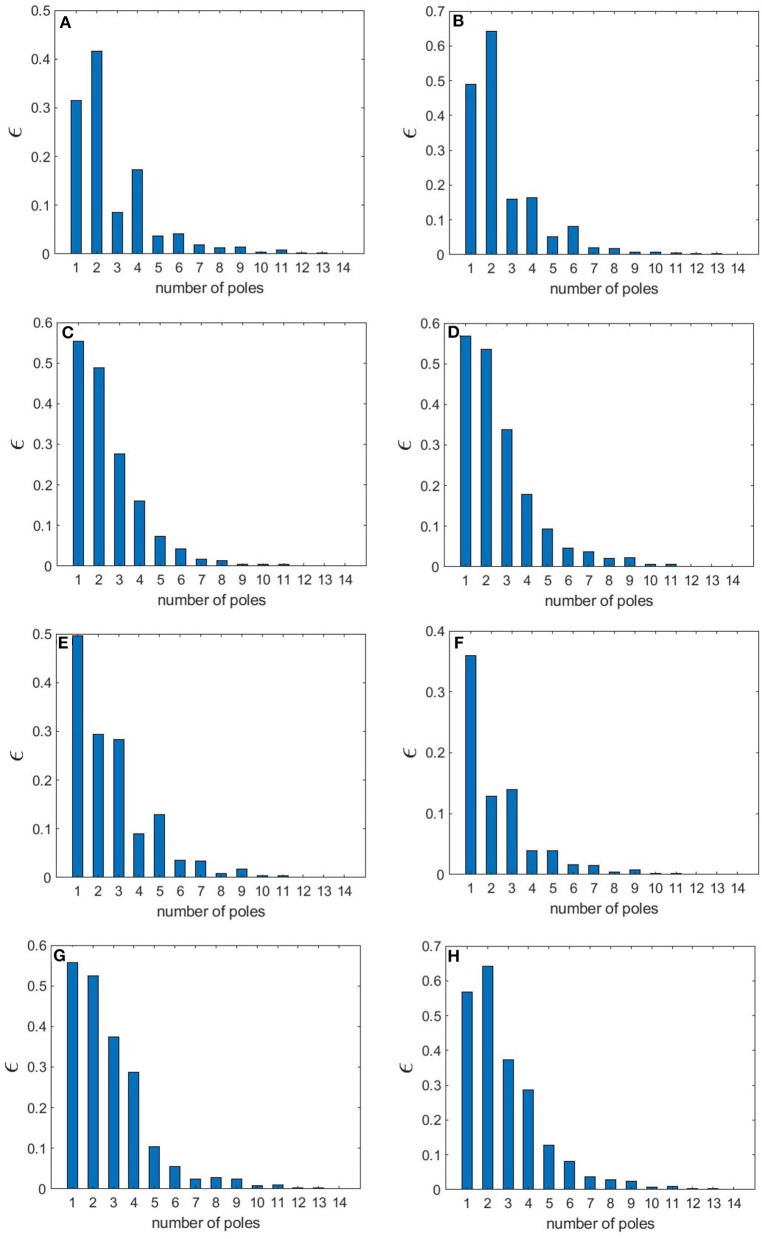
Root mean square error of the magnitude of the transfer function for different number of poles for various states. **(A)** EO state **(B)** EC state **(C)** REM state **(D)** S1 state **(E)** S2 state **(F)** SWS state **(G)** S2-Spindle state. In **(H)**, the highest ϵ value across all the states is shown vs. *n*.

The normalized rms error values corresponding to each sleep state shown in Table 3 are illustrated in Figure 12, where we observe how these values vary with the number of poles. We find that in the wake states. a 5-pole approximation is better than 6-pole approximation; this is due to the fact that the location of poles in a 5-pole approximation are approximately the same as for a 6-pole approximation (Figures 4A,B) except for the low frequency root which splits into two roots in the 6-pole approximation, reducing accuracy at low frequencies and increasing it at high frequencies (Figures 4A,B). In Figure 12, the rms error values ϵ of the magnitude of the transfer function vs. number of poles is shown for the different arousal states. In order to obtain a general number of poles to be used when studying any arousal state, we plot the worst cases of the maximum values of ϵ vs. the number of poles in Figure 12H across all the arousal states. We find that six poles are sufficient to attain better than 8% error, thereby preserving the main dynamics of the system.

**Table 3:** Root mean square error ϵ (in%) of the magnitude of the transfer function for each arousal state, where *n* is the number of poles.

***n***	**EO**	**EC**	**REM**	**S1**	**S2**	**SWS**	**Spindle**
1	32	49	55	57	50	36	57
2	42	64	49	54	29	13	52
3	9	16	28	34	28	14	0.37
4	17	16	16	18	8.9	3.9	2.9
5	3.7	5.1	7.3	9.4	13	3.9	10
6	4.2	8.2	4.2	4.6	3.6	1.7	5.5
7	2.0	1.9	1.8	3.7	3.4	1.5	2.4
8	1.3	1.9	1.4	2.0	0.85	0.42	2.8
9	1.5	0.67	0.41	2.2	1.7	0.74	2.4
10	0.38	0.70	0.40	0.62	0.42	0.20	0.80
11	0.78	0.59	0.39	0.58	0.40	0.19	0.93
12	0.18	0.22	0.10	0.09	0.06	0.04	0.24
13	0.21	0.22	0.08	0.09	0.06	0.04	0.23
14	0.09	0.17	0.06	0.02	0.01	0.02	0.11

*This table corresponds to the data illustrated in Figure 12*.

## 4. Summary and Discussion

We have bridged between physiologically-based NFT and experimental observations of brain resonances by decomposing the corticothalamic transfer function into spatial (eigenmodes) and temporal (poles) components. Modal-polar representation of NFT provides a unified platform to investigate a formula for ERPs and explore the number of poles needed to study the main dynamics of the system in each arousal state. Our main results are as follows:

- An approximation of the transfer function that describes the linear response of cortical brain activity to any input was achieved that allows it to be written as a ratio of polynomials. For each eigenmode, a pole of the transfer function corresponds to a given eigenfrequency or resonance. We restricted detailed analysis to the global mode ($k_{\eta }^{2}=0$), because it has uniform spatial properties across the two brain hemispheres. As a first approach, we applied the modal-polar expansion of the transfer function to derive a simpler formula for the ERP which is otherwise not analytically tractable. The calculations involved complex integrals that were solved via contour integration and the Cauchy-residue theorem.
- We validated the new expression of the ERP by comparing it with the exact ERP (calculated from NFT) for various states of arousal.
- We investigated how the poles for each arousal state shift as the approximation order increases and becomes more accurate. We found that as the approximation order increases, most poles move downwards in the complex plane, corresponding to these eigenfrequencies becoming more damped. For waking states, the least damped poles are mostly robust to these changes and adding more poles to the approximation involves shifts in the strongly damped poles, which contribute little to the overall dynamics. In these waking states, the roots corresponding to the alpha resonance shift slightly as we add more poles to the approximation, unlike the other sleep states. This is due to the significant alpha peak in the wake states, especially the EC which is characterized by its prominent alpha peak. For sleep states, especially REM and S1, we observed larger downward shifts in the poles toward more strongly damped eigenfrequencies, reflecting the smooth almost featureless spectra of these states.
- We found that six roots or three pairs of eigenfrequencies (poles) suffice to preserve the main dynamics of all arousal states to within ≈ 4% error. These roots correspond to the three resonances (low frequencies, alpha, and beta) and have been found to resemble a response expressed in terms of PID filters via control theory which is analogous to using a group of controllers in order to enhance the control system performance (Babaie-Janvier and Robinson, 2018).

Overall, this framework lays foundation for simplifying NFT results, and connecting NFT with observations of brain resonances, which in turn leads to an effective simplification of analysis of ERPs by expressing exact ERPs (based on NFT) in terms of those resonances. The present work has expanded previous work that approximated the transfer function and calculated poles for the waking state only (Gabay et al., 2018). Hence, this links observable electrophysiological resonances more directly to underlying dynamics and function, and yields interpretations of EEG resonances in terms of PID filters via control theory that provides insights on cognitive processes. The present analysis was restricted to the global mode, but future work could investigate the multimodal case as well as calculate other quantities from the transfer function such as coherence, correlation functions, and plasticity.

## Data Availability Statement

The original contributions presented in the study are included in the article/supplementary material, further inquiries can be directed to the corresponding author/s.

## Author Contributions

PR conceived the research. PR and RE-Z developed the theory, verified the analytical methods, and along with NG discussed all the results. NG wrote the computer code that was used and RE-Z added to this code, wrote the majority of the manuscript, and performed all the mathematical derivations. All authors contributed to the article and approved the submitted version.

## Conflict of Interest

The authors declare that the research was conducted in the absence of any commercial or financial relationships that could be construed as a potential conflict of interest.
